# When an incidental MRI finding becomes a clinical issue: Posterior lumbar subcutaneous edema in degenerative, inflammatory, and infectious conditions of the lumbar spine

**DOI:** 10.1007/s00508-019-01576-x

**Published:** 2019-11-26

**Authors:** Ursula Schwarz-Nemec, Klaus M. Friedrich, Michael A. Arnoldner, Felix K. Schwarz, Michael Weber, Siegfried Trattnig, Josef G. Grohs, Stefan F. Nemec

**Affiliations:** 1 Division of Neuroradiology and Musculoskeletal Radiology, Department of Biomedical Imaging and Image-guided Therapy, Medical University of Vienna, Waehringer Guertel 18–20, Vienna, Austria; 2 Department of Neurology, Medical University of Vienna, Vienna, Austria; 3 MR Center of Excellence, Department of Biomedical Imaging and Image-guided Therapy, Medical University of Vienna, Vienna, Austria; 4 Department of Orthopaedics and Trauma Surgery, Medical University of Vienna, Vienna, Austria

**Keywords:** Edema, Degeneration, Spondyloarthritis, Spondylodiscitis, Obesity

## Abstract

**Background:**

On magnetic resonance imaging (MRI), posterior lumbar subcutaneous edema (PLSE) is a frequent incidental, yet unclear finding within the deep subcutaneous perifascial tissue. This study aimed to investigate PLSE in various pathological lumbar conditions.

**Methods:**

This retrospective study included the MR images of the lumbar spine of 279 patients (age range 18–82 years) without cardiovascular, renal or hepatic diseases, 79 of whom had low-grade disc degeneration, 101 combined endplate and facet joint degeneration, 53 axial spondyloarthritis and 46 infectious spondylodiscitis. There were 232 patients with a body mass index (BMI) <30, and 47 with a BMI ≥30 (obese). For each group, the relationship between PLSE and BMI was analyzed using multiple logistic regression, and between PLSE extension and BMI using ordinal regression.

**Results:**

A PLSE was found in 11/79 (13.9%) patients with disc degeneration, 37/101 (36.6%) with endplate and facet joint degeneration, 7/53 (13.2%) with spondyloarthritis, and 28/46 (60.9%) with spondylodiscitis. For each group, a statistically significant relationship was demonstrated between PLSE and BMI (*P* = 0.000–*P* = 0.031), except for spondylodiscitis (*P* = 0.054), as well as between PLSE extension and BMI (*P* = 0.000–*P* = 0.049). A PLSE was found in 21.1% of nonobese and 72.3% of obese patients (*P* = 0.000).

**Conclusion:**

The presence of PLSE seems to be associated with various lumbar conditions, particularly in obese patients. Its perifascial location may suggest a potential fascial origin; however, PLSE should not to be confused with posttraumatic, postsurgical or infectious edema or edema associated with internal diseases.

## Introduction

Edema-like signal changes in the deep subcutaneous, perifascial soft tissue of the posterior lumbar spine or simply posterior lumbar subcutaneous edema (PLSE), are frequently depicted on routinely performed spinal magnetic resonance imaging (MRI) [1]. This edema is mostly reported as an incidental finding on MRI and has also been described on abdominopelvic computed tomography [1, 2]. Moreover, to date PLSE is a radiologically characterized term and finding rather than a clinically distinct entity. The origin of PLSE is not fully understood and remains unclear. In contrast, for obvious reasons, subcutaneous edema may occur as a sequela of spinal trauma, of previous surgical or interventional therapy, and in infectious infiltration. Clinically, local or generalized soft tissue edema may be associated with cardiac, renal, and hepatic diseases, malignant lymphadenopathy, venous thrombosis, allergic reactions, burns, or with certain drugs [3]. These refer to various pathophysiological mechanisms, including an increased capillary hydrostatic pressure, a decreased oncotic pressure, an increased capillary permeability, and lymphatic obstruction [3].

With respect to clinical parameters, studies have shown the association between PLSE and increased body weight, as well as an increased body mass index (BMI), and back fat thickness, suggesting a relationship between PLSE and obesity [1, 4–8]. These data confirm the anecdotal radiological experience that PLSE is commonly encountered in obese patients. The presence of PLSE seems to be associated with low back pain and posterior lumbar compartment degeneration, but has been found in asymptomatic, yet degeneratively changed subjects as well [9–11]. Since the available study data mainly refer to individuals with lumbar degeneration, the question arises whether PLSE is seen in any pathological lumbar condition and adult age group. The answer is becoming more important because the MRI findings of PLSE are usually disregarded in routine clinical practice and here, the changes of PLSE illustrate another important issue: MRI reveals vague findings, which may be inapparent to the patient and treating physician. This incidental observation, however, must be embedded in the clinical context, not least because edema often presents a stimulus for further diagnostic work-up to rule out various underlying conditions. Since the wording “incidental” in connection with an unclear etiology may somewhat insinuate insignificance, further investigation is a fortiori needed to associate the MRI presentation of PLSE with a clinical finding. Therefore, the purpose of this MRI study was to evaluate PLSE in patients with various lumbar conditions, such as in those with low-grade disc degeneration, combined Modic endplate degeneration and facet joint degeneration, axial spondyloarthritis, and infectious spondylodiscitis, and to study the relationship between these conditions and BMI.

## Material and methods

This retrospective study was approved by the institutional review board, and informed consent was waived.

### Study population

This study reviewed the medical records of lumbar spine MR examinations of 581 adult patients, performed within a 3-year period between January 2016 and December 2018. Initially, these patients were referred to the tertiary referral center to undergo an MRI work-up because of non-radicular low back pain. Inclusion criteria were: a) patients with low-grade disc degeneration, grade 2 or 3 according to the Pfirrmann classification [12], and without any other abnormalities, b) patients with combined Modic vertebral endplate degeneration (Modic types 1–3) and facet joint osteoarthitis, including possible additional disc degeneration and herniation according to the lumbar disc nomenclature of the North American Spine Society [12–15], c) patients with sero-negative, HLA B27-positive axial spondyloarthritis according to the Assessment of SpondyloArthritis international Society criteria, including lumbar involvement with or without sacroiliitis, as well as possible additional disc and vertrebral degeneration [16] and d) patients with bacterial, histologically proven, non-tuberculous spondylodiscitis, including possible additional disc and vertebral degeneration. Exclusion criteria included: vertebral fractures, spondylolysis, severe scoliosis and congenital vertebral abnormalities, Baastrup syndrome with interspinous fluid collection, edema of the paraspinal musculature or myositis, previous spinal trauma, previous spinal surgery or interventional therapy, spondylodiscitis with involvement of the posterior vertebral elements or with abscess formation, pregnancy and medical conditions, such as tumor diseases, venous thrombosis, cardiac, renal, hepatic and skin diseases. These stringent criteria were chosen to exclude potential sources of edema in the paravertebral soft tissues, changes associated with edema of the cutis into the subcutaneous fat tissue, and internal disorders that may produce local or generalized edema, anasarca, ascites or pulmonary congestion.

Based on the aforementioned criteria a total number of 279 study individuals were included and 302 patients were excluded. Of the 279 patients there were 79 patients with low-grade disc degeneration, 101 with combined Modic degeneration and facet joint degeneration, 53 with axial spondyloarthritis and 46 with infectious spondylodiscitis.

### MRI

All examinations of the lumbar spine were performed on a 3.0 T MR unit (Philips Achieva; Philips Medical Systems, Best, the Netherlands) using a 16-channel spine coil. All MR scans were performed with the patient in the supine position and head first on the scanner table. Sagittal MR images were oriented along the posterior edge of the vertebral bodies and covered the lumbar spine in a craniocaudal direction from the upper endplate of vertebral body Th12 to at least vertebral body S2. In the dorsoventral direction, the lumbar spine was visualized, including at least 5 cm of antevertebral soft tissue and the skin of the dorsum, and in the lateral direction, the lumbar spine was visualized including the transverse process on both sides. The standard MR protocol included the following sequence relevant to this study: a sagittal short tau inversion recovery (STIR) sequence (repetition time 4250–7000 ms, echo time 60–80 ms, flip angle 90°, slice thickness 3 mm, matrix 528 × 528 mm, duration ca. 3:58 min).

### Evaluation

All the MR examinations were anonymized and randomly presented on a picture archiving and communication system (IMPAX, Agfa HealthCare GmbH, Bonn, Germany) to an MRI specialist (S. F. N., 16 years experience in MRI), who was not aware of any patient data. For the assessment of the interreader agreement, a radiological fellow (U. S. N., 6 years experience in MRI) evaluated the same images in an independent reading session, who was also unaware of any patient data. For the assessment of the intrareader agreement, the image data were re-evaluated by the MRI specialist 4 weeks after the first evaluation.

**Fig. 1:**
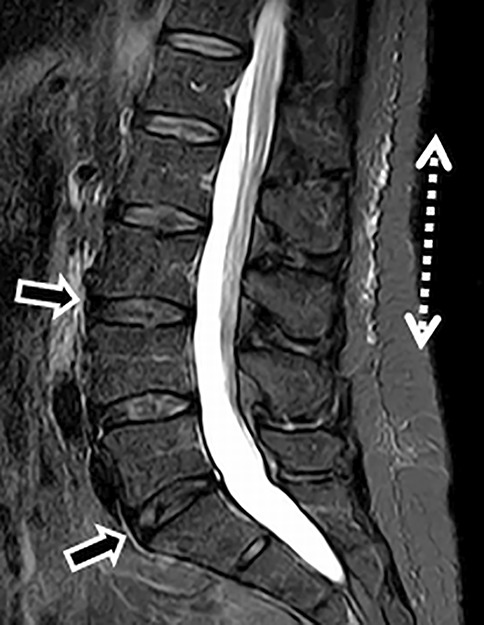
A 34-year-old male with multisegmental disc degeneration (*small arrows*) and posterior subcutaneous edema, which shows a multisegmental craniocaudal extension (*dotted arrow*)

The whole image stack of the sagittal STIR sequence was used to confirm or to rule out the presence of PLSE, which was defined as diffuse STIR-hyperintense signal changes within the deep posterior lumbar subcutaneous tissue that did not reach the superficial fat layers [1, 2, 4–11]. PLSE was also defined by its location along the fascia plane next to the thoracolumbar fascia and posterior to the spinous process, at the midline, and extending laterally [1, 2, 4–11]. Any edema was graded according to its craniocaudal extension with respect to the vertebral bodies from 1–5 (i.e., from one to five vertebral levels), and was consequently classified as monosegmental, one vertebral level, or multisegmental, two or more vertebral levels (Fig. 1).

### Statistical analysis

Statistical analysis was performed using software (IBM SPSS Statistics 25.0 for Windows; SPSS, Armonk, NY, USA). A *P*-value equal to or less than 0.05 was considered to indicate a significant difference. First, for each patient group the mean value, standard deviation, and range of the patients’ age and BMI, defined by the formula weight (kg)/height (m^2^), were recorded, and the differences were tested for statistical significance using a one-way analysis of variance. These demographic data were extracted from the patients’ MRI safety questionnaire. Second, the number and the percentage of female and male patients were recorded, and the differences were tested for statistical significance using a χ^2^-test. Third, for each patient group the number and percentage of obese (defined as BMI ≥30 kg/m^2^) and nonobese patients, with and without PLSE, were recorded using a cross-tabulation, and the overall differences were tested for statistical significance using Fisher’s exact test. Fourth, for each patient group a multiple logistic regression analysis was performed to test the relationship between PLSE and the patients’ BMI, including age and sex, for statistical significance. Fifth, an ordinal regression analysis (logit model) was performed to test the relationship between the PLSE extension and the patients’ BMI, including age and sex for statistical significance. Finally, the intrareader and interreader agreement for the assessment of PLSE was assessed using weighted κ statistics.

## Results

### Study population characteristics

The study population consisted of a total number of 279 individuals, including 124 females and 155 males (age range 18–82 years, mean age 49.8 years, mean weight 78.1 kg, weight range 41–125 kg). Table 1 gives details for each patient group, the patients’ age and BMI as well as the gender distribution. Statistically significant differences for the patients’ age (*P* < 0.001), BMI (*P* = 0.001), and sex (*P* = 0.002) were identified. Table 2 details the specific MRI findings for each patient group. Of the 79 patients with disc degeneration, 13 (16.5%) had monosegmental, and 66 (83.5%) multisegmental changes. Of the 101 patients with combined Modic and facet joint degeneration, 5 (5.9%) had monosegmental, and 96 (94.1%) multisegmental changes. Of the 53 patients with axial spondyloarthritis, 18 (34.0%) had monosegmental, and 35 (66.0%) multisegmental changes. Of the 46 patients with infectious spondylodiscitis, 43 (93.5%) had monosegmental, and 3 (6.5%) multisegmental changes.

**Table 1:** Descriptive statistics for patients’ age, BMI, and gender distribution

				***N***	**Mean**		**Standard deviation**		**Range**	
*Age (Y)*		*Disc degeneration*		79	39.82		11.80		18–75	
		*Modic and facet joint degeneration*		101	58.34		13.23		24–82	
		*Axial spondyloarthritis*		53	35.62		8.81		18–58	
		*Infectious spondylodiscitis*		46	64.65		12.52		18–81	
–		*Total*		279	49.82		16.48		18–82	
*BMI*		*Disc degeneration*		79	25.20		3.61		15.43–41.52	
		*Modic and facet joint degeneration*		101	27.31		5.41		19.00–48.83	
		*Axial spondyloarthritis*		53	24.55		3.84		16.62–34.72	
		*Infectious spondylodiscitis*		46	25.19		4.67		14.20–35.83	
–		*Total*		279	25.84		4.67		14.20–48.83	
–	–		***N***	**Female (*N*)**		**Male (*N*)**		**Female (%)**		**Male (%)**
*Sex*	*Disc degeneration*		79	30		49		38.0		62.0
	*Modic and facet joint degeneration*		101	60		41		59.4		40.6
	*Axial spondyloarthritis*		53	18		35		34.0		66.0
	*Infectious spondylodiscitis*		46	16		30		34.8		65.2
–	*Total*		279	124		155		44.4		55.6

*Y* Years; *BMI* body mass index; *N* number

**Table 2:** Specific MRI findings for each patient group

	*N*	Findings *N* (%)
Disc degeneration	79	Pfirrmann grade 2: 26 (32.9)
		Pfirrmann grade 3: 53 (67.1)
*Modic and facet joint degeneration*	101	Modic type 1: 7 (6.9)
		Modic type 2: 48 (47.5)
		Modic type 3: 38 (37.6)
		Modic type 2/3: 8 (7.9)
		Disc abnormalities and facet joint degeneration: 101 (100)
*Axial spondyloarthritis*	53	Vertebral lesions only: 9 (17.0)
		Vertebral lesions and sacroiliitis: 44 (83.0)
		Additional disc and/or vertebral degeneration: 53 (100)
*Infectious spondylodiscitis*	46	Additional disc and/or vertebral degeneration: 46 (100)

*N* Number

### Findings of PLSE and the relationship between PLSE and demographic parameters

**Fig. 2:**
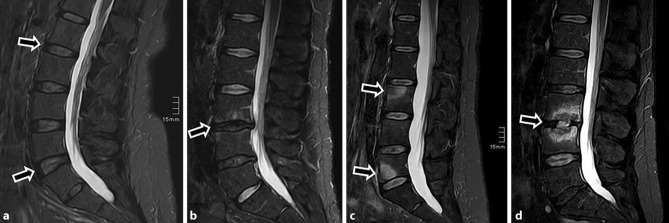
**a**–**d**: Four different patients without posterior subcutaneous edema: **a** a 50-year-old male (BMI 23.9) with disc degeneration Pfirrmann grade 2 (*arrows*), **b** a 32-year-old male (BMI 26.7) with combined Modic type I and facet joint degeneration (*arrow*), **c** a 32-year-old female (BMI 23.7) with axial spondyloarthritis (*arrows*) and **d** a 57-year-old male (BMI 27.4) with infectious spondylitis (*arrow*)

**Fig. 3:**
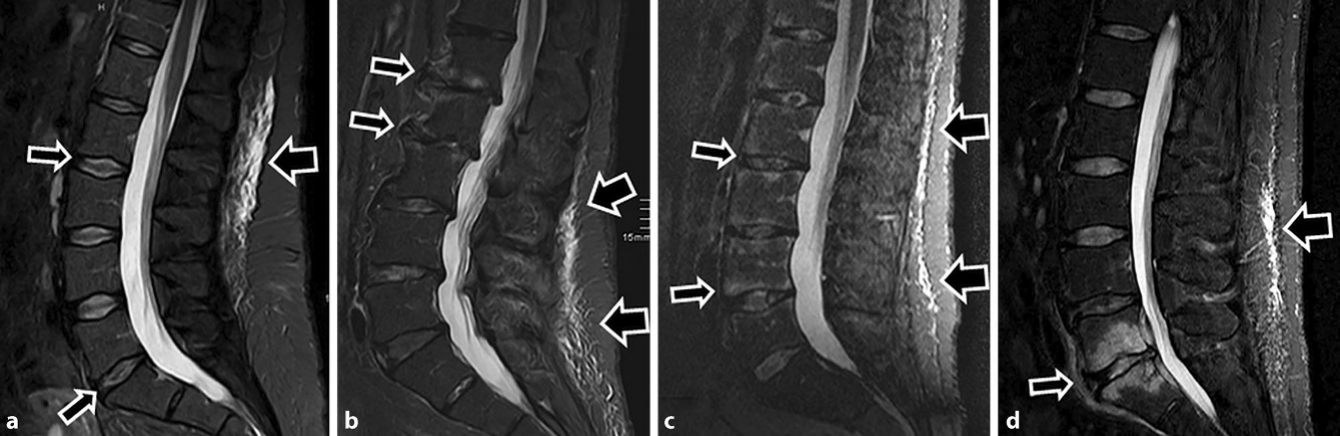
**a**–**d**: Four different patients with multisegmental posterior subcutaneous edema (*large arrows*): **a** a 34-year-old female (BMI 32.4) with disc degeneration Pfirrmann grade 2 (*small arrows*), **b** a 77-year-old female (BMI 28.4) with combined Modic type I and facet joint degeneration (*small arrows*), **c** a 42-year-old male (BMI 25.9) with axial spondyloarthritis (*small arrows*) and **d** a 57-year-old male (BMI 28.4) with infectious spondylitis (*small arrow*)

Overall, PLSE was found in 83/279 (29.7%) patients and was not depicted in 196/279 (70.3%) patients (Fig. 2 and 3). In detail, PLSE was found in 11/79 (13.9%) patients with low-grade disc degeneration, in 37/101 (36.6%) with combined Modic and facet joint degeneration, in 7/53 (13.2%) with axial spondyloarthritis, and in 28/46 (60.9%) with infectious spondylodiscitis (Table 3). Furthermore, PLSE was found in 21.1% of nonobese and in 72.3% of obese patients (*P* = 0.000) (Table 3). Of a total of 83 patients with PLSE, only 4 (4.8%) patients demonstrated a monosegmental PLSE extension. The vast majority, 79/83 patients (95.2%), demonstrated a multisegmental PLSE extension as follows: 10/11 (90.9%) in disc degeneration, 34/37 (91.9%) in Modic and facet joint degeneration, 7/7 (100%) in axial spondyloarthritis and 28/28 (100%) in infectious spondylodiscitis. For each group, there was a statistically significant relationship between PLSE and the BMI (*P* = 0.000–*P* = 0.031), except for infectious spondylodiscitis (*P* = 0.054) (Table 4). For each group, there was a statistically significant relationship between the PLSE extension and the patients’ BMI (*P* = 0.000–*P* = 0.049) (Table 4). There was no statistically significant relationship between PLSE and its extension and the patients’ age and sex, except for the patients’ age in combined Modic and facet joint degeneration (*P* = 0.013) (Table 4).

**Table 3:** Descriptive statistics for PLSE in obese and nonobese patients

	BMI	Patients *N* (%)	No PLSE *N* (%)	Present PLSE *N* (%)
*Disc degeneration*	*BMI <30*	74 (93.7)	67 (90.5)	7 (9.5)
	*BMI ≥30*	5 (6.3)	1 (20.0)	4 (80.0)
	*Total*	79 (100)	68 (86.1)	11 (13.9)
*Modic and facet joint degeneration*	*BMI <30*	75 (74.3)	58 (77.3)	17 (22.7)
	*BMI ≥30*	26 (25.7)	6 (23.1)	20 (76.9)
	*Total*	101 (100)	64 (63.4)	37 (36.6)
*Axial spondyloarthritis*	*BMI <30*	45 (84.9)	42 (93.3)	3 (6.7)
	*BMI ≥30*	8 (15.1)	4 (50.0)	4 (50.0)
	*Total*	53 (100)	46 (86.8)	7 (13.2)
*Infectious spondylodiscitis*	*BMI <30*	38 (82.6)	16 (42.1)	22 (57.9)
	*BMI ≥30*	8 (17.4)	2 (25.0)	6 (75.0)
	*Total*	46 (100)	18 (39.1)	28 (60.9)
*Total*	*BMI <30*	232 (83.2)	183 (78.9)	49 (21.1)
	*BMI ≥30*	47 (16.8)	13 (27.7)	34 (72.3)
	*Total*	279 (100)	196 (70.3)	83 (29.7)

*BMI* body mass index ; *N* number; *PLSE* posterior lumbar subcutaneous edema

**Table 4:** Relationship between PLSE and its extension and the patients’ BMI, age, and sex

***–***			***P*** **value**	**Odds ratio**
*Disc degeneration*	*PLSE*	*Age*	0.950	0.998
		*BMI*	0.003	1.529
		*Sex*	0.692	1.397
*Modic and facet joint degeneration*	*PLSE*	*Age*	0.019	1.057
		*BMI*	0.000	1.359
		*Sex*	0.541	1.393
*Axial spondyloarthritis*	*PLSE*	*Age*	0.278	0.936
		*BMI*	0.031	1.303
		*Sex*	0.420	0.389
*Infectious spondylodiscitis*	*PLSE*	*Age*	0.524	1.018
		*BMI*	0.054	1.167
		*Sex*	0.474	1.661
–			***P*** **value**	**Estimate**
*Disc degeneration*	*Extension*	*Age*	0.735	0.011
		*BMI*	0.004	0.262
		*Sex*	0.798	−0.199
*Modic and facet joint degeneration*	*Extension*	*Age*	0.013	0.048
		*BMI*	0.000	0.241
		*Sex*	0.776	0.130
*Axial spondyloarthritis*	*Extension*	*Age*	0.402	−0.048
		*BMI*	0.049	0.225
		*Sex*	0.397	−1.009
*Infectious spondylodiscitis*	*Extension*	*Age*	0.209	0.035
		*BMI*	0.002	0.215
		*Sex*	0.897	0.077

*BMI* body mass index; *PLSE* posterior lumbar subcutaneous edema

### Reader agreement

For the assessment of PLSE, there was excellent intrareader (weighted κ = 1) and interreader agreement (weighted κ = 1).

## Discussion

This study assessed PLSE in patients with slight and marked lumbar degeneration, sero-negative inflammation, and infection, as well as the relationship between PLSE and the patients’ BMI. Different frequencies of PLSE were observed in patients who were referred for MRI because of low back pain: in ~14% of patients with disc degeneration, in ~37% with advanced lumbar degeneration, in ~13% with axial spondyloarthritis, and in ~61% with spondylodiscitis. It could be argued that the high frequency of PLSE in spondylodiscitis is attributable to its infectious nature compared to disc degeneration; however, focal PLSE within the deep fatty tissue did not exhibit any visible infectious spread from the paraspinal or vertebral structures, since posterior spondylitic involvement and abscess formation were a priori excluded. Also, as previously shown a direct phlegmonous spread seems unlikely because the degree of infectious spondylitis was not correlated with the degree of PLSE [7].

For the vast majority, there was no statistically significant relationship between PLSE and its extent, and the patients’ age and sex, based on separate calculations for each pathological group. This methodology was chosen because of the multiple associations between the different conditions and the demographic parameters that result in statistical multicollinearity [17]. The latter impedes the interpretation as to what extent the different frequencies of PLSE are influenced by the condition itself or by the covariates. Moreover, it should be considered that the studied conditions do involve different demographics per se with statistically significant differences in age, sex, and BMI. For example, spondyloarthritis is seen in young adults (mean age ~36 years) and spondylodiscitis occurs in older adults (mean age ~65 years), with the latter linked to a much higher PLSE frequency. In addition, there is an overlap of conditions since older patients with spondylodiscitis, in particular, will also be affected by lumbar degeneration.

For the vast majority, there was a statistically significant relationship between PLSE and its extent and the patients’ BMI. Because of this relationship, which has also been demonstrated in previous studies, several authors have formulated hypotheses to provide explanations for the occurrence of PLSE in obesity [1, 4–10]. This includes various cascades of pathophysiological changes on a macrovascular, microvascular, hormonal, and molecular levels [1, 4–10]. It has been also hypothesized that lumbar degeneration and low back pain may be associated with a decrease in venous and lymphatic drainage from soft tissues due to the restriction of physical activity [9]. This consideration was supported by the finding that PLSE was particularly seen in hospitalized patients with prolonged bed rest [4]. Furthermore, to explain PLSE in obesity and older individuals, co-existing internal conditions, such as cardiac, hepatic, or renal dysfunction, have been discussed, which are frequently associated with forms of edema [5, 7]. Notably, for example, in cardiac failure, typical peripheral edema is located in the dependent areas, particularly in the lower extremities in ambulatory patients; the edema, however, may shift to the back and sacral area in bedridden patients [3]. Therefore, to avoid a clinical overlap, in the present study patients with internal conditions that may produce edema or generalized fluid retention were excluded.

On MRI, PLSE is best observed on a sagittal midline view within the deep-fat tissue along the thoracolumbar fascia, covering a triangle-shaped area on transverse images [1, 7, 9, 10]. The MRI findings of PLSE seem to mimic a peritendinitis-like appearance, potentially suggesting abnormal stress and overuse of the fascial tissues. A well-recognized example of peritendinitis is found at the Achilles tendon that is surrounded by loose connective tissue, called paratenon, without having a synovial sheath. Here, tendon overuse may cause an inflammatory reaction in the paratenon known as peritendinitis [18]. Early peritendinitis is seen as edema along the normal tendon; subsequently, it may co-exist with a pathological tendon, including changes in signal intensity and shape that may progress to a partial rupture [18]. The presence of PLSE is also reminiscent of a commonly observed prepatellar subcutaneous edema that is associated with body weight and chronic microtrauma, as well as of peritrochanteric edema, both of which should not be mistaken for actual tendinitis [19–21]. The PLSE, as well as prepatellar and peritrochanteric edema, may be seen in conjunction with degeneration, in patients with and without pain symptoms [19–21]. Although this retrospective study was not able to assess the thoracolumbar fascial complex itself, PLSE might be related to potential pathophysiological changes in connective tissue layers and fascial derangement, which are found in patients with low back pain [22–24]. The imaging of fascial abnormalities, for instance, of necrotizing or plantar fasciitis, has been well documented and subsequently established in daily practice, whereas ultrasound or MRI data have rarely included myofascial back pain [25, 26]. Thus, this issue will require much more future research to be conducted, particularly to correlate the imaging findings with the patients’ symptoms.

This retrospective study has several limitations. First, this study included a total of 279 adult patients with various lumbar conditions but with a limited number of individuals in each group. With respect to the relationship between PLSE and the BMI in spondylodiscitis, there was a statistical tendency (*P* = 0.054) but no significant result, which may be attributable to the number of patients in this group (*n* = 46). Second, because it was not possible to compare the patients with age-matched asymptomatic normal individuals, the actual specific disease-related influence on the presence of PLSE remains unclear. Such a comparison, however, may be virtually impossible because of lumbar aging, and thus, degeneration, from the second decade of life onward [27, 28]. Third, although male gender and android fat distribution have been identified as risk factors for Modic degeneration, within the study ~60% of patients with Modic changes were females, which may reflect institutional MRI habits [29]. Fourth, the retrospective PLSE assessment was based on highly fluid-sensitive, sagittal STIR sequences alone, but did not include transverse T2-weighted and STIR sections with complete lumbar coverage [4, 11]. Consequently, the signal intensity, thickness, and length of the thoracolumbar fascia could not be sufficiently assessed, which may potentially provide further evidence about the origin of edema, as suggested in this study. Finally, the data obtained from adults do not permit statements to be made about potential PLSE in the pediatric population. As a side note there is anecdotal evidence based on prenatal MRI to diagnose fetal abnormalities that PLSE also occurs as an incidental finding in young healthy pregnant individuals. This will be addressed by an upcoming investigation at this MRI research facility.

In conclusion, PLSE seems to be associated with various degenerative, inflammatory and infectious conditions of the lumbar spine, particularly in obese patients. The frequency of PLSE may be influenced by these various conditions, albeit the actual PLSE to condition relationship remains unclear. Although further validation of these results must await future investigations to more clearly elucidate the origin of PLSE, its perifascial MRI presentation may suggest a potential fascial origin. Therefore, it is suggested terming these findings lumbar perifascial edema rather than subcutaneous edema. On a routine basis, it should not be confused with soft tissue edema in a posttraumatic or postsurgical setting or in infection and should also be differentiated from local or generalized edema associated with specific conditions, such as cardiovascular, renal, or hepatic diseases. Ultimately, lumbar perifascial edema develops from an imaging finding to a clinical finding within the clinical spectrum of edema that should not be disregarded.
